# Mitochondrial ROS and Mitophagy in Intervertebral Disc Degeneration: Mechanistic Interplay and Links to Regulated Cell Death

**DOI:** 10.3390/ijms27219628

**Published:** 2026-10-28

**Authors:** Lice Wang, Kai Cao, Haozhe Cheng, Zhong Fang

**Affiliations:** Department of Orthopedics, Tongji Hospital, Tongji Medical College, Huazhong University of Science and Technology, Wuhan 430030, China; wanglice97@hust.edu.cn (L.W.); kaibox5@163.com (K.C.)

**Keywords:** intervertebral disc degeneration, mitochondrial reactive oxygen species, mitophagy, mitochondrial quality control, regulated cell death, oxidative stress, nucleus pulposus

## Abstract

Intervertebral disc degeneration (IVDD) develops through interacting mechanical, matrix, nutritional, inflammatory and age-related processes. Mitochondrial reactive oxygen species (mtROS) and mitophagy participate in some of these contexts, but current evidence does not place mitophagy failure at the start of every degenerative trajectory. Most mechanistic studies have been performed in nucleus pulposus (NP) cells. In these models, mechanical overload, inflammatory mediators and oxidant stress can depolarize mitochondria, disturb fission–fusion balance and increase mtROS. Mitophagy can limit injury when damaged cargo is selected and delivered through a competent lysosomal pathway; accumulation of pathway markers during blocked flux, or mitochondrial clearance that is not matched by renewal, need not be protective. The most extensive mechanistic literature concerns apoptosis and senescence. Evidence linking mitochondrial quality control to pyroptosis and ferroptosis is growing but remains model-dependent, whereas direct evidence for necroptosis is comparatively sparse. We use a capacity-matching framework to organize these findings around damage burden, cargo selection, lysosomal completion and mitochondrial renewal within the regional metabolic constraints of the disc. This is an interpretive framework rather than a newly identified pathway. Its purpose is to define testable conditions under which mitophagy is likely to be protective, insufficient or maladaptive, while preserving the distinction between NP-derived evidence and the more limited evidence from annulus fibrosus and cartilage endplate cells.

## 1. Introduction

Intervertebral disc degeneration involves progressive proteoglycan loss, disruption of the collagen network, dehydration, annulus fibrosus (AF) fissures, cartilage endplate (CEP) sclerosis or calcification, and declining cellularity. These changes impair load distribution and accompany several painful spinal disorders, although structural degeneration does not necessarily produce pain. The three disc regions have distinct but interdependent functions. The gelatinous NP resists compression, the fibrous AF contains the NP and bears tensile load, and the CEP regulates solute exchange with the vertebral circulation. A mechanistic account of IVDD must therefore connect cellular stress with matrix turnover, nutrient transport and communication among these regions.

Mitophagy dysfunction should not be interpreted as a necessary starting point for IVDD. Cartilage-endplate calcification can restrict solute transport, annulus fibrosus fissuring can compromise nucleus pulposus containment, matrix abnormalities can weaken load bearing, and age-related proteoglycan depletion can reduce hydration even when mitochondrial clearance remains competent [1,2,3,4,5,6,7,8,9,10,11,12,13,14,15,16]. These processes may secondarily impose mitochondrial stress, but they establish that mitophagy is a context-dependent modifier among several interacting mechanisms rather than a universal initiating event.

Mitochondrial dysfunction connects these features of the disc microenvironment with changes in cell fate and matrix metabolism. Mature NP cells rely mainly on glycolysis, but their mitochondria remain important for redox signaling, calcium buffering, metabolite exchange and innate immune responses. Degenerative human disc tissue and experimental IVDD models show loss of membrane potential, permeability-transition-pore opening, abnormal fission or fusion, reduced ATP production and increased mtROS [1,2,3,4,5,6,7]. Mitochondria are both a source and a target of these oxidants. Low ROS levels contribute to adaptive signaling, whereas sustained production damages mitochondrial proteins, lipids and DNA. Injury to the respiratory chain can then generate further ROS and prolong the stress response.

Mitophagy selectively delivers damaged or superfluous mitochondria to lysosomes. In the canonical damage-sensing pathway, loss of mitochondrial membrane potential stabilizes PINK1 on the outer membrane. PINK1 then recruits Parkin, promotes ubiquitination of mitochondrial proteins and engages autophagy receptors. Hypoxia-responsive and membrane-associated receptors, including BNIP3, BNIP3L/NIX, FUNDC1, OPTN and PHB2, provide additional routes to mitochondrial sequestration. In the chronically hypoxic, acidic and nutrient-limited disc, these pathways contribute to normal metabolic adaptation as well as stress responses [17].

mtROS production and mitophagy influence each other. Mitochondrial damage can initiate quality control, and completed mitophagy removes organelles that would otherwise continue to generate ROS. IVDD studies, however, do not support the assumption that greater pathway activation is always beneficial. Compression increases PINK1 and Parkin but can block mitophagosome–lysosome fusion [18]. Loss of NLRX1 produces widespread mitochondrial fragmentation and compensatory PINK1–Parkin activity associated with senescence [19]. BNIP3 deficiency increases mitochondrial delivery through alternative receptors without restoring mitochondrial morphology or NP-cell metabolism [17]. High compression and BNIP3-linked excessive autophagy have also been associated with senescent or apoptotic phenotypes [20,21]. Protein abundance and autophagosome number alone therefore cannot show whether mitochondrial clearance is complete, selective or protective. This review does not propose a new mitochondrial pathway or a universal theory of IVDD. Instead, it examines mitochondria-involved disease contexts by integrating damage burden, cargo selection, lysosomal completion and mitochondrial replacement within the metabolic and mechanical constraints of the disc.

## 2. Mitochondrial Redox Homeostasis and Mitophagy in the Intervertebral Disc

### 2.1. A Specialized Redox Environment

The healthy adult disc contains few blood vessels, and most nutrients reach its cells by diffusion across the CEP. Oxygen and glucose concentrations decline toward the NP, whereas lactate accumulates and lowers extracellular pH. NP cells are consequently adapted to low oxygen tension through HIF-dependent transcription and glycolytic metabolism. This adaptation does not eliminate mitochondrial function. Networked mitochondria are present in NP cells, and their morphology and metabolic contribution are actively regulated under hypoxia [17,22]. Mitochondria also provide signals that couple energy state to matrix synthesis, inflammatory responses and survival.

Redox homeostasis reflects a balance among ROS production, antioxidant buffering, repair and removal of damaged macromolecules or organelles. Electron leakage from respiratory-chain complexes, altered NADH/NAD+ balance, calcium overload, permeability transition and excessive fission can all increase mitochondrial superoxide. Superoxide is converted to hydrogen peroxide, which can diffuse and signal more broadly. Antioxidant systems—including superoxide dismutase, glutathione, thioredoxin and Nrf2-dependent enzymes—normally constrain this signal. SIRT3 further supports mitochondrial redox fitness by deacetylating mitochondrial metabolic and antioxidant proteins. SIRT3 expression is reduced in more severely degenerated human NP tissues, while SIRT3 loss increases TBHP-induced mtROS, membrane-potential loss, senescence and apoptosis [23]. HIF-1α can also restrain mitochondrial oxidative stress in disc cells, consistent with the idea that hypoxic adaptation is part of normal redox control rather than simply a pathological response [24].

### 2.2. Mitophagy as One Arm of Mitochondrial Quality Control

Mitophagy operates within a larger quality-control network comprising mitochondrial proteostasis, biogenesis, fission, fusion and lysosomal degradation. Mildly damaged mitochondria may be repaired or fused with healthier organelles. Fission can segregate a damaged segment, which is then selected for removal, while mitochondrial biogenesis replaces lost capacity. Productive mitophagy, therefore, requires more than initiation; damaged cargo must be recognized, enclosed, delivered to an acidified lysosome and degraded, after which the mitochondrial network must recover.

PINK1–Parkin is the most frequently examined pathway in IVDD. Parkin loss aggravates mitochondrial depolarization, mtROS accumulation and apoptosis in stressed NP cells, whereas Parkin-dependent clearance is associated with preserved matrix metabolism [25,26,27,28]. Downstream cargo recognition also matters. TBK1-dependent phosphorylation of p62 at Ser403 promotes selective autophagy, and TBK1 overexpression protects NP cells from TNF-α-induced apoptosis and senescence in a Parkin- and autophagy-dependent manner [29]. Mitofusins further connect network configuration with cargo selection, although Mfn2-associated autophagy and Mfn1-associated quality-control phenotypes should not be treated as interchangeable [30,31].

Receptor-mediated pathways add context specificity. OPTN increases mitolysosome formation and limits mtROS, senescence and matrix catabolism in oxidant-stressed NP cells [32]. FUNDC1 expression can be transcriptionally enhanced by a circFUNDC1–CDK9 complex, promoting mitochondrial clearance in human NP cells and explants [33]. BNIP3 is especially relevant under hypoxia, but its role extends beyond acting as an LC3-binding receptor: BNIP3 helps maintain mitochondrial morphology, glycolytic capacity and ATP production [17]. PHB2, located in the inner mitochondrial membrane, has recently been linked to removal of damaged mitochondria and suppression of necroptosis [34]. These pathways partly overlap, and compensation among receptors means that loss of one receptor does not necessarily reduce total mitophagy.

Available evidence does not show that NP cells use all mitophagy pathways equally. PINK1–Parkin is the most frequently examined route after depolarization caused by compression, oxidants or inflammatory cytokines [18,25,26,27,28,29]. BNIP3 appears particularly relevant to chronic hypoxic and metabolic adaptation [17,22], whereas FUNDC1 and OPTN have mainly been studied under oxidant or inflammatory stress [32,33]. Evidence for PHB2 is currently confined to a selected necroptosis model [34]. These routes can overlap or compensate for one another, and no comparative study establishes a single obligatory pathway across healthy, early-degenerate and advanced NP cells. Apparent pathway preference should therefore be interpreted according to the stressor, cell state and flux assay used.

### 2.3. Disc Region, Cell State and Metabolic Context

Disc regions differ in oxygen supply, substrate use and mechanical demand. Direct measurements in human disc cells found higher oxygen consumption in AF than NP cells, and AF cells can oxidize lactate released by NP cells, supporting region-dependent metabolic coupling [35,36]. The CEP regulates solute transport into the disc; oxidative injury, ferroptosis and calcification in endplate chondrocytes can therefore affect both local cell survival and nutrient delivery to the inner disc [8,9,10,11,12,13]. These regional differences make it inappropriate to treat a response measured in isolated NP cells as a mechanism operating uniformly throughout the disc.

The evidence base remains NP-dominant, but AF evidence is not absent. Oxidative-stress-induced ferroptotic changes have been reported in both AF and NP cells [37], and an AF-repair study linked engineered-exosome treatment to restoration of mitophagy and matrix homeostasis in AF models [38]. Such studies broaden the regional evidence without establishing that AF and NP cells use identical mitochondrial programs. CEP work is concentrated on oxidative injury, ferritinophagy or ferroptosis, Parkin-associated mitophagy and calcification [8,9,10,11,12,13]. Because head-to-head comparisons of NP, AF and CEP cells are uncommon, the review reports the tissue source of each finding and does not assign a dominant death pathway to a disc region.

Cell state further shifts the balance between repair and disposal. NP-derived progenitors, mature NP cells and senescent cells differ in proliferative demand, mitochondrial mass and lysosomal reserve. Restoration of SIRT1/FOXO3-associated flux lowers ROS and apoptosis in NP-derived mesenchymal stem cells [39,40,41,42,43]. The optimal turnover rate for a progenitor, however, may not apply to a terminally differentiated or already senescent cell.

Metabolic and ionic conditions further shape this response. NLRX1 interacts with SLC39A7 to regulate mitochondrial Zn^2+^ trafficking, OPA1 processing, selective fission and mitophagy. Loss of NLRX1 causes network collapse, widespread fragmentation and compensatory PINK1–Parkin activation [19]. BNIP3 deficiency alters substrate use, reduces glycolytic capacity and ATP production, and causes early degenerative changes despite increased clearance through alternative receptors [17]. Acidic pH, nutrient deprivation, advanced glycation products and disturbed mitochondria–ER contacts also alter redox load and the capacity to recover after turnover [44,45,46,47,48,49,50]. ATF5- and Sesn2-associated mitochondrial unfolded-protein responses provide an upstream repair mechanism that cooperates with mitophagy when proteostasis is insufficient [51,52]. Mitophagy activity therefore cannot be interpreted independently of the metabolic state of the disc cell or the replacement capacity of the remaining mitochondrial network.

### 2.4. Mitochondrial-Derived Vesicles as a Complementary Quality-Control Route

Mitochondrial-derived vesicles (MDVs) provide a selective response to spatially restricted mitochondrial damage without requiring elimination of the entire organelle. These single- or double-membrane carriers can enrich oxidized mitochondrial proteins and deliver selected cargo to endolysosomal compartments through routes that are partly independent of ATG5 and LC3. Specific MDV populations are regulated by PINK1–Parkin and use Syntaxin-17-dependent fusion machinery [53,54,55,56]. MDVs may therefore operate upstream of organelle-scale mitophagy by excising damaged subdomains while preserving the remaining mitochondrial network. In non-disc models, increased MDV trafficking can also partly compensate for impaired LC3-mediated mitophagy [57]. Mitochondrial cargo can additionally enter extracellular vesicle pathways, although intracellular, lysosome-directed MDVs and extracellular mitochondria-derived vesicles represent functionally distinct destinations [58,59].

Evidence from degenerative human and experimental NP cells now links MDV-related transport to IVDD. Cytoplasmic mislocalization of the RNA-binding protein TDP43 was accompanied by mitochondrial accumulation of the protein, loss of mitochondrial membrane potential and cellular senescence. TDP43 was also enriched in extracellular vesicle fractions released by degenerative or IL-1β-stimulated NP cells. Depletion of TOMM22-positive vesicles reduced this cargo, supporting a mitochondrial origin. The vesicles induced inflammatory and senescent phenotypes in recipient NP cells, whereas genetic or pharmacological suppression of TDP43 reduced mitochondrial dysfunction, senescence and disc degeneration [60]. In this setting, vesicular transport may export mitochondrial stress rather than confining quality control to intracellular disposal.

The intracellular and extracellular routes require different experimental evidence. Intracellular lysosome-directed MDVs are defined by mitochondrial budding, enrichment of selected mitochondrial cargo and delivery to LAMP1-positive endolysosomal compartments without extracellular release [53,54,55,56]. Extracellular vesicles containing mitochondrial material require isolation from conditioned medium, particle and topology characterization, demonstration of mitochondrial cargo and assessment of established extracellular-vesicle markers and contaminants. TOMM22, TOMM20 or VDAC1 support mitochondrial origin but do not, by themselves, establish vesicle biogenesis or distinguish an extracellular MDV from another extracellular vesicle carrying mitochondrial proteins [58,59,60]. The IVDD study therefore supports extracellular transfer of mitochondria-associated TDP43 from degenerative NP cells, whereas the STX17/SNAP29/VAMP7-dependent lysosomal route is drawn from foundational non-disc studies.

**Figure 1:**
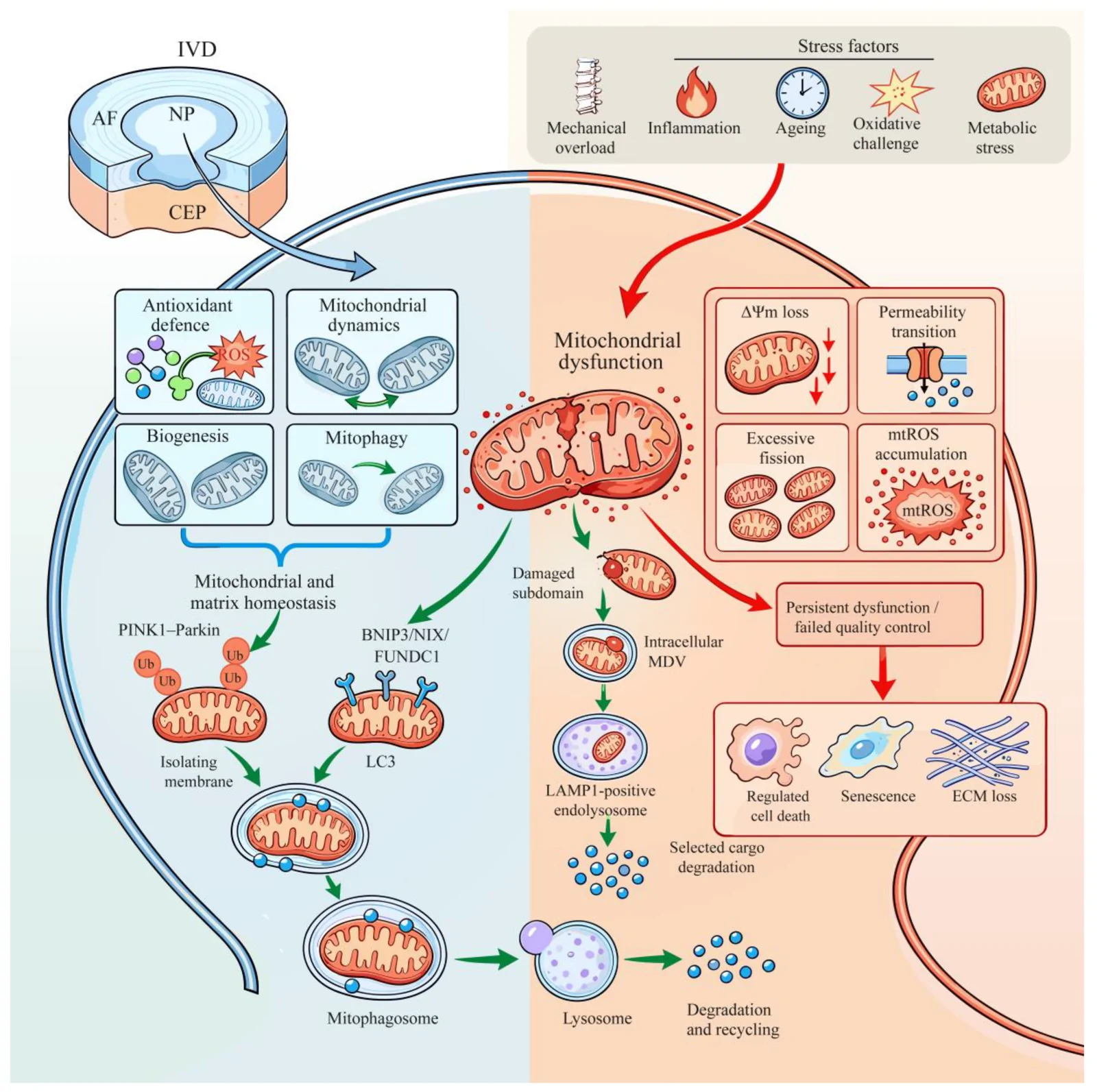
Mitochondrial quality control in nucleus pulposus cells. The disc inset identifies the NP cell represented in the main panel. IVDD-associated stressors can cause mitochondrial membrane-potential loss, permeability transition, excessive fission and mtROS accumulation. Antioxidant defense, mitochondrial dynamics, biogenesis and mitophagy contribute to mitochondrial and matrix homeostasis. Damaged mitochondria can enter PINK1–Parkin- or receptor-mediated mitophagy. Smaller intracellular MDVs may deliver selected cargo to LAMP1-positive endolysosomes; this lysosome-directed route is established mainly outside the disc field. Persistent mitochondrial dysfunction can contribute to regulated cell death, senescence and ECM loss, but the scheme does not imply that mitophagy failure is required for all IVDD. AF, annulus fibrosus; BNIP3, BCL2-interacting protein 3; CEP, cartilage endplate; ECM, extracellular matrix; FUNDC1, FUN14 domain-containing protein 1; IVD, intervertebral disc; LAMP1, lysosome-associated membrane glycoprotein 1; LC3, microtubule-associated protein 1 light chain 3; MDV, mitochondrial-derived vesicle; mtROS, mitochondrial reactive oxygen species; NIX, BCL2-interacting protein 3-like; NP, nucleus pulposus; PINK1, PTEN-induced kinase 1; ROS, reactive oxygen species; Ub, ubiquitin; ΔΨm, mitochondrial membrane potential. Green arrows indicate mitochondrial clearance or homeostatic recovery, whereas red arrows indicate persistent mitochondrial injury and degenerative outcomes; blue elements denote the broader mitochondrial quality-control network.

MDV formation may therefore have different consequences within the disc. Packaging damaged mitochondrial constituents could preserve functional mitochondrial mass in the donor cell, whereas extracellular release may expose neighboring cells to harmful cargo. It is not yet known whether TDP43-containing vesicles share their biogenesis with lysosome-directed MDVs, increase when mitophagic flux is saturated or selectively carry oxidized mitochondrial components. MDVs are best regarded as a complementary branch of mitochondrial quality control rather than a substitute for mitophagy. Their effect in IVDD is likely to depend on cargo identity, intracellular or extracellular destination, uptake by recipient cells and clearance from the disc [53,54,55,56,57,58,59,60].These mitochondrial quality-control routes and their potential outcomes are summarized in Figure 1.

## 3. Stress-Dependent Coupling of mtROS Production and Mitophagy in IVDD

### 3.1. Mechanical Loading and Mitochondrial Damage

Mechanical loading is necessary for disc health, but excessive magnitude, duration or frequency can exceed cellular adaptation. In a bovine whole-disc one-strike model, a single high-energy deformation rapidly decreased mitochondrial membrane potential and produced an early, sustained rise in ROS. MitoQ markedly reduced this signal, supporting a mitochondrial origin, and also attenuated apoptosis- and necroptosis-associated changes and subsequent matrix damage [61]. Human-cell and CEP studies likewise connect matrix stiffness or excessive compression to Piezo1 signaling, mitochondrial fission and disturbed autophagy/mitophagy [62,63,64,65,66,67,68,69]. Although acute organ injury does not reproduce chronic human IVDD, this temporal sequence places mitochondrial redox disruption near the beginning of the degenerative response.

Sustained compression shows why increased mtROS and mitophagy initiation may coexist with continuing injury. In human NP cells, high compression increases mitochondrial permeability transition, depolarization and fission, decreases fusion, and recruits PINK1 and Parkin. Lysosomal dysfunction nevertheless prevents mitophagosome–lysosome fusion, leaving damaged mitochondria in place despite the rise in initiation markers [18]. High-magnitude compression also suppresses SIRT1 and induces ROS, mitochondrial dysfunction and senescence; protection by SIRT1 requires PINK1 [70]. HIF1A-, MIF- and SIRT3-linked programs preserve autophagic or mitochondrial adaptation under compression, whereas loss of these reserves favors apoptosis and oxidative injury [71,72,73]. One study, however, found that reducing PINK1–Parkin activity attenuated p53/p21-dependent senescence [20]. More recent work links an adaptive mechanical response to ITGB1/vimentin-dependent PINK1–Parkin activity [74]. Differences in loading protocol, cell source, exposure time and flux measurement probably explain part of this disagreement. The same pathway may indicate effective clearance at one stage and overwhelmed or maladaptive turnover at another.

Mechanical transduction also determines which mitochondrial material becomes available for clearance. In CEP cells, Piezo1-driven Ca^2+^/CaMKII/Drp1 signaling promotes excessive fission, mitochondrial dysfunction, inflammation, apoptosis and senescence [75]. ERK1/2-dependent Drp1 activation similarly fragments mitochondria and is associated with apoptosis and pyroptosis in oxidant-stressed NP cells [76]. Neither study directly measured mitophagic flux. They nevertheless show how fission may either isolate damaged segments for disposal or fragment the network too extensively for quality control to remain effective.

### 3.2. Inflammation, Oxidative Challenge and Ageing-Associated Stress

Inflammatory cytokines, such as TNF-α and IL-1β, increase ROS, impair mitochondrial function and stimulate matrix-catabolic pathways. TNF-α-induced Parkin-dependent mitophagy is initially protective; silencing Parkin increases mtROS, membrane-potential loss and apoptosis, whereas enhancing the pathway supports survival [25]. TBK1 and OPTN provide additional links between inflammatory stress, selective autophagy and mitochondrial cargo handling [29,32]. However, inflammation can also damage lysosomes and autophagosome maturation, converting increased initiation into incomplete turnover.

Hydrogen peroxide and TBHP are widely used to model oxidative stress. They reproducibly induce mitochondrial depolarization, mtROS, apoptosis, senescence and matrix degradation. These models allow causal testing of Nrf2–SIRT3, DJ-1–AKT–HK2–Parkin, the ATF5-dependent mitochondrial unfolded-protein response and FUNDC1 [33,51,77,78,79,80,81,82,83]. High-glucose/AGE conditions, circadian disruption and ASIC1a activation provide metabolically distinct routes to mitochondrial redox failure [84,85,86]. Bolus oxidants are nevertheless broad and are often applied under normoxic, nutrient-rich culture conditions. They establish pathway competence but cannot reproduce the sequence operating over decades of human degeneration.

Aging imposes a slower and more persistent mitochondrial stress. Progerin accumulation in NP cells impairs mitochondrial dynamics and promotes apoptosis and senescence [87]. Declining proteostasis, lysosomal acidity and autophagic flux allow damaged mitochondria to accumulate, while senescent cells expose neighboring cells to cytokines and oxidants [88]. EGR1 suppression and engineered restoration of AMPK–FOXO1a-associated mitophagy reduce mitochondrial injury and senescence in recent models [89,90]. The response also changes over time. DJ-1, AKT and HK2 support Parkin recruitment during early stress, but prolonged stress depletes DJ-1 and permits mitochondrial apoptosis [78]. Oxidative injury and mitochondrial clearance therefore become progressively uncoupled as adaptive reserves decline.

## 4. Bidirectional Interactions Between mtROS and Mitophagy

### 4.1. mtROS and Depolarization Initiate Mitochondrial Surveillance

Oxidative damage to respiratory complexes, cardiolipin and mitochondrial DNA lowers membrane potential and increases permeability transition. Depolarization blocks normal PINK1 import and degradation, allowing PINK1 to accumulate on the outer membrane and recruit Parkin. Oxidative and hypoxic signaling can also activate receptor-mediated pathways. In disc cells, early Parkin induction, DJ-1/HK2 recruitment, BNIP3 translocation and FUNDC1 activation are consistent with mtROS-containing stress acting upstream of mitochondrial selection [17,22,25,33,78].

mtROS should nevertheless not be treated as a single quantitative trigger. The location, duration and molecular target of ROS matter. A transient oxidant signal may initiate antioxidant transcription and selective clearance, whereas widespread lipid and protein oxidation can disable autophagy proteins, lysosomal membranes or the cytoskeleton required for cargo transport. ROS may therefore stimulate mitophagy initiation while simultaneously reducing the probability that flux will be completed.

### 4.2. Completed Mitophagy Limits mtROS and Danger Signaling

Direct manipulation of mitophagy supports its role in mitochondrial redox control. Parkin silencing increases mtROS and apoptosis during inflammatory or oxidative stress [25,26]. OPTN overexpression increases mitolysosome formation and reduces MitoSOX fluorescence and senescence, whereas OPTN deficiency has the opposite effects [32]. Nrf2–SIRT3 activation improves mitochondrial clearance, antioxidant capacity and survival; genetic interference or autophagy inhibition weakens these benefits [77]. Taurine, urolithin A, hyaluronic acid, platelet lysate and stromal-cell extracellular vesicles also reduce mitochondrial injury when flux is restored [91,92,93,94,95]. Because these interventions have several biological targets, their efficacy cannot by itself establish a single mitophagy-dependent mechanism.

Removing damaged mitochondria also restricts secondary danger signals. Persistent permeability transition can release mitochondrial DNA into the cytosol, where it activates TLR9 or cGAS–STING signaling. Humanin preserves mitochondrial homeostasis, lowers mtROS and mtDNA leakage and suppresses mtDNA–TLR9–NF-κB signaling during ferroptotic stress [96]. Taurine has been reported to reduce cytosolic mtDNA and enhance autophagic degradation of STING, thereby suppressing TBK1–IRF3/NLRP3 signaling in NP progenitor cells [97]. These studies do not directly establish selective mitophagy as the only mechanism, but they connect mitochondrial redox control and organelle clearance with sterile inflammation [98].

### 4.3. Why Increased Mitophagy Markers May Coexist with Degeneration

Increased pathway markers do not necessarily indicate increased mitophagic flux. PINK1, Parkin, LC3-II, p62 and autophagosomes may accumulate because initiation has risen, degradation is blocked or damaged cargo exceeds clearance capacity. Compression-induced lysosomal failure provides the clearest IVDD example: PINK1–Parkin recruitment increases while mitophagosomes fail to fuse with functional lysosomes [18]. Curcumin restores general autophagic flux by improving autophagosome–lysosome fusion and lysosomal function, with corresponding reductions in mitochondrial injury, apoptosis, senescence and ECM loss [99]. The study did not isolate mitochondria-specific cargo throughout, but it shows why the terminal degradative step must be measured.

Cargo selectivity is another determinant of outcome. NLRX1 supports fission of damaged mitochondrial segments. Without NLRX1, widespread fragmentation generates abundant cargo and compensatory PINK1–Parkin activity associated with severe senescence [19]. BNIP3 deletion increases mitochondrial delivery through alternative receptors but does not restore morphology or metabolism [17]. In other settings, excessive BNIP3-linked activity has been associated with YAP1-dependent pyroptosis or senescence, apoptosis and mTOR-sensitive disc injury [21,100,101]. NDUFA4L2 perturbation likewise shows that reduced mitochondrial turnover can be protective in a defined redox context [102]. Turnover may therefore become harmful when cargo selection is poor, clearance exceeds biogenesis or mitophagy forms part of a broader pro-death program.

Taken together, these studies support a capacity-matching interpretation for IVDD models in which mitochondrial stress is demonstrable. The component processes—damage sensing, selective disposal, lysosomal degradation and mitochondrial biogenesis—are established parts of mitochondrial quality control. The contribution of the present framework is its joint interpretation under the unusual metabolic and mechanical conditions of the disc, not the proposal of a new molecular pathway. It predicts that increasing PINK1–Parkin initiation will not rescue compressed cells if mitophagosome–lysosome fusion remains blocked; that accelerating clearance may be detrimental when mitochondrial replacement and ATP reserve are inadequate; and that the same intervention may differ among NP, AF and CEP cells because their stress exposure and metabolic reserve differ. These predictions require direct testing and should not be read as evidence that mitophagy failure is necessary or sufficient for IVDD.

**Figure 2:**
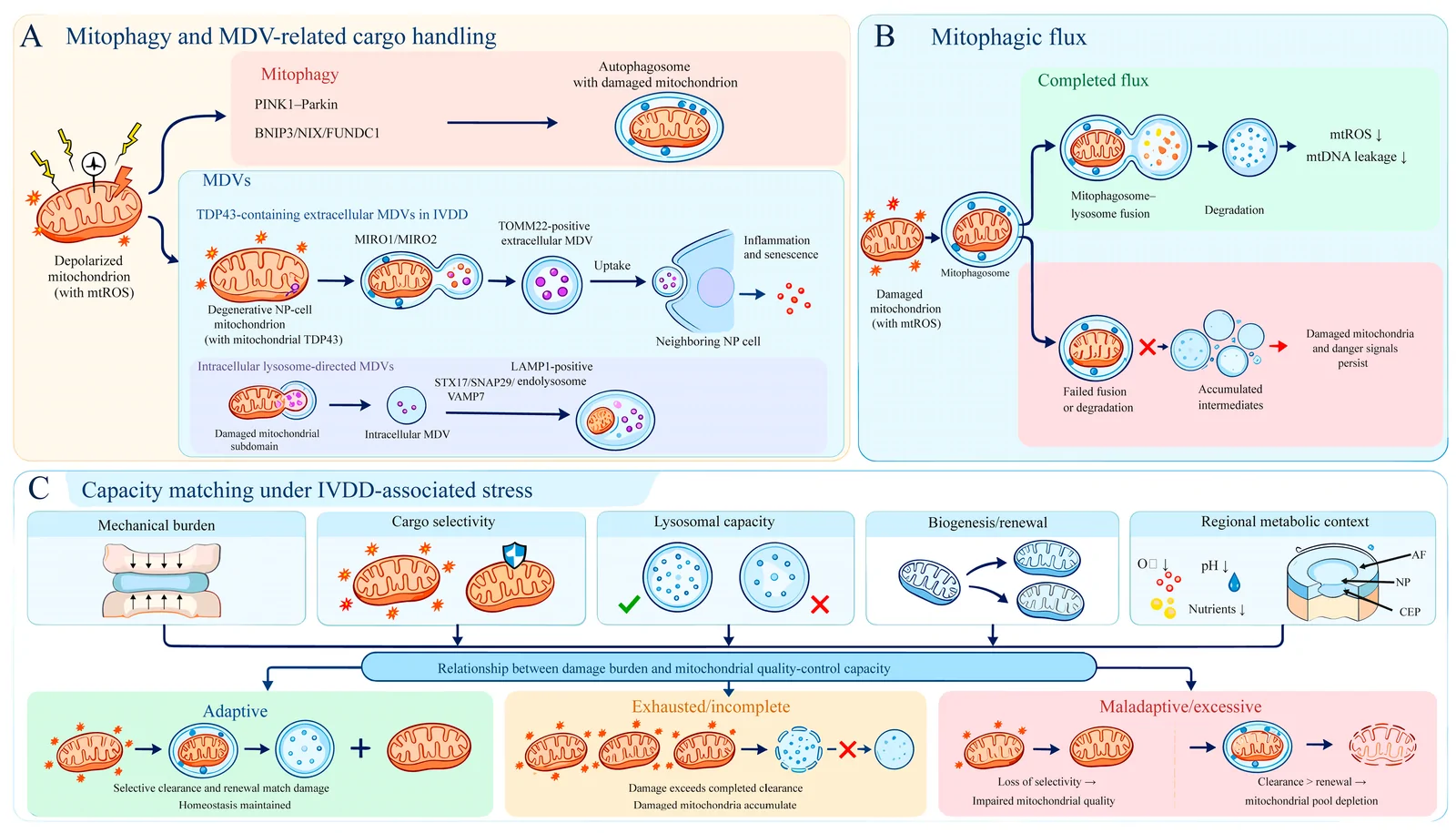
Mitophagic flux, MDV-related cargo handling and the capacity-matching framework. (**A**) Depolarized mitochondria may undergo PINK1–Parkin- or receptor-mediated mitophagy or generate vesicles with different destinations. One IVDD study identified TDP43-containing, TOMM22-positive extracellular vesicles of mitochondrial origin that were taken up by neighboring NP cells and promoted inflammatory and senescent phenotypes [60]. The intracellular STX17/SNAP29/VAMP7-dependent route to LAMP1-positive endolysosomes is based mainly on non-disc studies [53,54,55,56]. (**B**) Completed mitophagic flux requires lysosomal fusion and cargo degradation; accumulation of intermediates can instead indicate blocked flux. (**C**) The authors’ framework relates damage burden to cargo selectivity, lysosomal capacity, mitochondrial renewal and regional metabolic context. The three illustrated outcomes are hypotheses for experimental testing, not established stages of IVDD. AF, annulus fibrosus; BNIP3, BCL2-interacting protein 3; CEP, cartilage endplate; FUNDC1, FUN14 domain-containing protein 1; IVDD, intervertebral disc degeneration; LAMP1, lysosome-associated membrane glycoprotein 1; MDV, mitochondrial-derived vesicle; MIRO1/2, mitochondrial Rho GTPase 1/2; mtDNA, mitochondrial DNA; mtROS, mitochondrial reactive oxygen species; NIX, BCL2-interacting protein 3-like; NP, nucleus pulposus; PINK1, PTEN-induced kinase 1; SNAP29, synaptosomal-associated protein 29; STX17, syntaxin 17; TDP43, TAR DNA-binding protein 43; TOMM22, translocase of outer mitochondrial membrane 22; VAMP7, vesicle-associated membrane protein 7. Arrows indicate the direction of cargo handling or pathway progression; green checks denote completed clearance, whereas red crosses denote blocked or incomplete steps.

This integration yields three testable predictions. First, increasing PINK1–Parkin initiation should not protect compressed cells when lysosomal fusion remains defective. Second, accelerating clearance should become maladaptive when biogenesis and ATP reserve cannot replace the removed organelles. Third, an intervention should produce different outcomes in NP, AF and CEP cells, or at early and late disease stages, when their stress burden and mitochondrial reserve differ. These predictions distinguish the framework from a generic assumption that more mitophagy is beneficial and require flux and functional measurements rather than pathway-protein abundance alone.The distinction between completed and blocked mitophagic flux, together with MDV-related cargo handling, is illustrated in Figure 2. IVDD-associated stressors and the corresponding mitochondrial responses are summarized in Table 1.

**Table 1:** IVDD-associated stressors and mitochondrial quality-control responses.

IVDD-Associated Stressor	Mitochondrial Alteration	Mitophagy Response	Region/Model Emphasis and Consequence	Representative Studies
Mechanical overload	Membrane-potential loss, permeability transition, excessive fission and mtROS accumulation	PINK1–Parkin initiation may increase, whereas sustained loading can impair lysosomal completion	NP cells, acute whole-disc and selected CEP models; apoptosis, senescence and matrix catabolism	Huang et al. [20]; Rannou et al. [16]; Li et al. [61]; Ke et al. [62]; Zheng et al. [63]; Wang et al. [72]
Inflammatory stress	TNF-α and IL-1β increase mitochondrial injury and mtROS	Parkin, TBK1–p62 and OPTN support clearance when downstream flux remains competent	Predominantly NP-cell and rodent models; reduced survival and matrix synthesis	Zhang et al. [25]; Hu et al. [29]; Shin et al. [31]; Wang et al. [103]; Zhao et al. [104]; Ma et al. [105]
Oxidant exposure	TBHP or H_2_O_2_ causes depolarization, mtROS accumulation and mitochondrial proteotoxic stress	Nrf2–SIRT3, FUNDC1, ATF5 and DJ-1–Parkin pathways promote stress adaptation	Predominantly NP cells, with selected CEP studies; apoptosis, senescence and matrix loss	Wang et al. [23]; Zhang et al. [25]; Gu et al. [33]; Kang et al. [10]; Hu et al. [77]; Lin et al. [78]
Hypoxic, metabolic and ionic stress	Altered substrate use, ATP production, Zn^2+^ trafficking and OPA1 processing	BNIP3 and NLRX1 coordinate metabolic adaptation, selective fission and mitochondrial turnover	Mainly NP-cell models; loss of NP phenotype and early degeneration	Madhu et al. [17]; Song et al. [19]; Madhu et al. [22]; Yang et al. [24]; Hua et al. [44]; Liu et al. [45]
Ageing-associated stress	Damaged mitochondria accumulate as antioxidant and lysosomal capacity declines	Incomplete or excessive turnover can sustain mtROS and the senescence-associated secretory phenotype	Human disc/NP and rodent evidence; persistent inflammatory and matrix-catabolic signalling	Nasto et al. [7]; Hu et al. [32]; Xu et al. [87]; Wu et al. [89]; Wang et al. [106]; Feng et al. [107]
MDV-mediated communication	TDP43 accumulates in damaged mitochondria and is exported in TOMM22-positive vesicles	MDV export may remove cargo from donor cells but transmit stress to neighbouring NP cells	One IVDD-specific NP study plus foundational non-disc MDV biology; intercellular inflammation and senescence	Soubannier et al. [53]; Soubannier et al. [54]; McLelland et al. [55]; Towers et al. [57]; Todkar et al. [58]; Liao et al. [60]

Representative studies are shown rather than an exhaustive evidence list. Evidence is predominantly NP-derived unless another source is stated. Liao et al. [60] provides IVDD-specific evidence for MDV-mediated communication; references [53,54,55,56,57,58,59] establish the underlying MDV biology. AGE, advanced glycation end product; ATF5, activating transcription factor 5; FUNDC1, FUN14 domain-containing protein 1; IL-1β, interleukin-1β; IVDD, intervertebral disc degeneration; MDV, mitochondrial-derived vesicle; mtROS, mitochondrial reactive oxygen species; NLRX1, NLR family member X1; NP, nucleus pulposus; OPA1, optic atrophy 1; OPTN, optineurin; PINK1, PTEN-induced kinase 1; SIRT3, sirtuin 3; TBHP, tert-butyl hydroperoxide; TBK1, TANK-binding kinase 1; TDP43, TAR DNA-binding protein 43; TNF-α, tumor necrosis factor α; TOMM22, translocase of outer mitochondrial membrane 22.

## 5. mtROS, Mitophagy and Regulated Cell Death

### 5.1. Apoptosis

Among the regulated cell-death programs considered here, apoptosis has the strongest causal support. Mitochondrial depolarization, permeability transition, Bax/Bcl-2 imbalance, cytochrome-c release and caspase activation recur in AF, NP and progenitor-cell models [16,108,109]. Parkin knockdown aggravates mtROS accumulation, mitochondrial dysfunction and apoptosis [25,26]. Melatonin-induced clearance reduces TBHP-induced apoptosis, and interference with Parkin or mitophagy weakens this effect [110]. The miR-182-5p/SIRT1–circERCC2 axis provides further genetic evidence connecting mitochondrial turnover with apoptosis [111].

Several adaptive pathways modify this relationship [83]. SIRT1 requires PINK1 to limit compression-induced mitochondrial dysfunction and senescence [70]. Nrf2–SIRT3 improves antioxidant defense, autophagic flux and resistance to apoptosis [77]. ATF5-dependent mitochondrial proteostasis cooperates with PINK1-mediated clearance [51], whereas DJ-1–AKT–HK2 promotes early Parkin recruitment but loses efficacy during prolonged stress [78]. MANF–MFN2 signaling also connects mitochondrial fusion and mitophagy with apoptosis, although the evidence is currently limited to cultured human NP cells [112]. Across these models, competent mitochondrial quality control consistently raises the threshold for intrinsic apoptosis.

Mitophagy is unlikely to be the sole mediator of these effects. Most compounds that enhance mitochondrial clearance also alter redox, inflammatory or metabolic pathways. Reversal by cyclosporin A, chloroquine or 3-methyladenine indicates autophagy dependence but not mitochondrial selectivity, because each inhibitor has broader actions. Genetic perturbation, flux reporters, and direct measurement of mitochondrial cargo provide stronger evidence. Even with these limitations, bidirectional manipulation of Parkin and several upstream pathways makes apoptosis the best-supported death outcome jointly associated with mtROS and mitophagy.

### 5.2. Pyroptosis and Inflammasome-Associated Death

mtROS, oxidized mitochondrial components and leaked mtDNA can activate NLRP3. In primary human NP cells, reducing mtROS suppresses NLRP3 signaling and improves matrix metabolism [81,98,103,104,113,114]. SIRT1-, CREG1- and calreticulin-associated PINK1–Parkin activity has likewise been linked to less inflammasome activation or pyroptosis [105,115,116]. These findings support a mitochondrial damage–inflammasome connection, but inflammasome activation alone is not equivalent to pyroptotic death.

Attribution to pyroptosis requires caspase-1 activation, gasdermin cleavage, membrane permeabilization and pathway-specific rescue. ERK1/2–Drp1-dependent fragmentation activates both pyroptotic and apoptotic markers, and Drp1 inhibition improves both outcomes [76]. A20, PTPN22 and taurine connect improved mitochondrial turnover with reduced gasdermin- or caspase-1-associated injury, whereas PRDM1 provides a transcriptional route to CASP1 activation [117,118,119,120]. Melatonin-mediated Parkin activity has also been associated with lower inflammasome signaling in a radiculopathy model [121]. Removal of damaged mitochondria may therefore raise the pyroptotic threshold, but this evidence is newer and less independently replicated than the evidence for apoptosis.

### 5.3. Ferroptosis

Ferroptosis is driven by iron-dependent phospholipid peroxidation and failure of glutathione–GPX4 defense. mtROS can promote lipid oxidation, while mitochondrial metabolism, iron handling and membrane state influence susceptibility. In CEP chondrocytes, Nrf2/HO-1 activation increases Parkin-associated mitophagy and reduces oxidative dysfunction, ferroptosis and calcification [8,9,122]. Changes in mitochondrial redox state and clearance at the endplate may therefore affect nutrient supply to the entire disc as well as CEP-cell survival.

Several recent NP-cell studies provide more direct evidence [68,69,123,124]. Loss of VMP1 impairs PINK1–Parkin activity, worsens mitochondrial dysfunction and increases ferroptosis-related changes [125]. FBXO2 combines mitochondrial clearance with LCN2 degradation, thereby limiting iron overload, glutathione depletion and lipid peroxidation [126]. HIF-1α/BNIP3 and FBXW7 studies also place mitophagy upstream of ferroptosis resistance. ACSL1, NOX4–LRRC8A and metallothionein-2A identify lipid-metabolic, inflammatory and antioxidant pathways that may converge on mitochondrial redox stress [127,128,129,130,131]. Mitophagy may thus reduce one source of oxidative pressure without correcting every component of ferroptosis.

Humanin reduces erastin-induced ferroptosis and preserves membrane potential. It also limits permeability transition, mtROS, mtDNA leakage and HSP27-sensitive NF-κB and JAK2/STAT3 signaling [96]. Mitochondrial homeostasis is clearly involved, but mitophagic flux was not the principal causal variable. TBHP creates a related interpretive problem because it is used as both an oxidative-stress and a ferroptosis inducer, despite activating apoptosis and other injury programs. Ferroptosis should therefore be assigned using lipid peroxidation and iron measurements, GPX4 or glutathione changes, characteristic rescue and exclusion of competing death modes. Evidence that mtROS and mitophagy jointly regulate ferroptosis is increasing, but the precise relationship remains incompletely defined.

### 5.4. Necroptosis

Necroptosis is mediated by RIPK1–RIPK3–MLKL signaling and produces inflammatory membrane rupture. Acute one-strike loading increases necroptosis-associated changes in whole bovine discs, and MitoQ or necrostatin-1 partially reduces cell loss and matrix injury [61]. This places mtROS upstream of a necroptotic component but does not itself establish mitophagy control.

The clearest evidence connecting mitophagy with necroptosis comes from PHB2 manipulation. In human NP cells exposed to TNF-α/SM-164/Z-VAD-FMK, PHB2 loss worsens mitochondrial dysfunction and RIPK1–RIPK3–MLKL signaling. PHB2 overexpression restores autophagosome–lysosome activity, limits necroptosis and preserves matrix proteins [34]. Compression-induced death of NP progenitors provides related evidence, although broad HSP90 inhibition and mixed apoptosis or necroptosis limit pathway specificity [109]. Recent reviews accordingly regard necroptosis as a less developed branch of IVDD research than apoptosis or pyroptosis [132].

### 5.5. Senescence and Mixed Cell-Fate States

Senescence is not regulated cell death, but it is a major alternative fate after mitochondrial stress. Senescent NP cells remain in the tissue and release inflammatory and matrix-catabolic factors, allowing degeneration to progress without immediate loss of cellularity. PINK1-, OPTN- and FUNDC1-associated clearance generally reduces ROS and senescence [32,33,67,70,82,106,107,133]. NLRX1 deficiency produces a different outcome: widespread fragmentation and compensatory mitophagy accompany a pronounced senescent phenotype [19]. Whether a damaged cell dies or persists in a senescent state may depend on residual ATP, lysosomal competence and the capacity to rebuild a functional mitochondrial network.

Markers of more than one cell-fate program are often detected in the same IVDD model, but this does not establish simultaneous execution or a fixed sequence of transitions. For example, A20-associated enhancement of mitophagy reduced both pyroptotic and apoptotic readouts in NP cells, and inhibition of pyroptosis also reduced apoptosis in that model [117]. This result supports pathway interaction, but it does not define a universal switch between pyroptosis and apoptosis. Similar caution applies when mtROS reduction improves several endpoints at once: a shared upstream lesion may influence multiple programs without determining which program executes death in each cell. The evidence base and principal limitations for each cell-fate programme are summarized in Table 2.

**Table 2:** Evidence base and interpretive limits for mitochondrial involvement in cell-fate programs in IVDD.

Cell-Fate Programme	Defining Evidence	mtROS–Mitophagy Connection	Evidence Base and Principal Limitation	Representative Studies
Apoptosis	Depolarization, cytochrome-c release and caspase activation	Competent Parkin- or receptor-mediated clearance generally limits mitochondrial apoptosis	Bidirectional pathway manipulation in human disc-derived cells, organ culture and rodent models; much of the intervention literature remains pleiotropic	Zhang et al. [25]; Lan et al. [26]; Hu et al. [77]; Xu et al. [102]; Chen et al. [110]; Xie et al. [111]
Pyroptosis	Caspase-1 activation, gasdermin cleavage and IL-1β/IL-18 release	mtROS and leaked mtDNA promote NLRP3 signalling; improved clearance can suppress this response	NP-cell and rodent pathway studies with gasdermin/caspase readouts; inflammasome activation is still sometimes used as a surrogate for execution	Lu et al. [98]; Wang et al. [103]; Ma et al. [105]; Zhang et al. [115]; Song et al. [116]; Ou et al. [119]
Ferroptosis	Lipid peroxidation, iron accumulation and impaired GSH–GPX4 defence	Mitochondrial dysfunction promotes lipid oxidation; PINK1–Parkin, BNIP3, VMP1 and FBXO2 are linked to resistance	NP and CEP biochemical, inhibitor and rescue studies; literature is recent and direct human causal evidence is limited	Zhu et al. [96]; Liu et al. [123]; Dou et al. [124]; Zhang et al. [125]; Wu et al. [126]; Wang et al. [127]
Necroptosis	RIPK1–RIPK3 activation, MLKL phosphorylation and membrane rupture	MitoQ-sensitive mtROS can precede necroptosis, and PHB2 links mitochondrial clearance to pathway restraint	Acute whole-disc and selected NP-cell studies; few independent replications and little human validation	Zhu et al. [34]; Li et al. [61]; Hu et al. [109]; Zhang et al. [132]
Senescence	Stable growth arrest, p16/p21 induction and a senescence-associated secretory phenotype	PINK1-, OPTN- and FUNDC1-associated clearance is usually protective, but maladaptive fragmentation reveals context dependence	Multiple NP-cell and in-vivo studies; regional comparisons are limited and senescence is a non-lethal fate	Song et al. [19]; Hu et al. [32]; Gu et al. [33]; Chen et al. [82]; Wu et al. [89]; Wang et al. [106]

This table is a descriptive evidence map, not a formal GRADE assessment. The evidence base was characterized by model coverage, pathway-specific perturbation or rescue, execution-specific readouts and independent replication. Senescence is a persistent non-lethal cell fate rather than regulated cell death. Co-detection of markers does not establish simultaneous execution or a transition between programs. ACSL1, acyl-CoA synthetase long-chain family member 1; AF, annulus fibrosus; CEP, cartilage endplate; FBXO2, F-box protein 2; FUNDC1, FUN14 domain-containing protein 1; GPX4, glutathione peroxidase 4; GSH, glutathione; IVDD, intervertebral disc degeneration; LCN2, lipocalin 2; MLKL, mixed lineage kinase domain-like protein; mtROS, mitochondrial reactive oxygen species; NP, nucleus pulposus; OPTN, optineurin; PHB2, prohibitin 2; PINK1, PTEN-induced kinase 1; RIPK, receptor-interacting protein kinase; VMP1, vacuole membrane protein 1.

### 5.6. Shared Upstream Determinants and Limits of Current Evidence

Hypoxia, ATP depletion, mitochondrial depolarization, lysosomal dysfunction, inflammasome priming, iron availability and lipid-peroxidation defense can alter susceptibility to apoptosis, pyroptosis, ferroptosis or senescence. The corresponding execution machinery is distinct: mitochondrial outer-membrane permeabilization and caspases support apoptosis; caspase-1 and gasdermin cleavage support pyroptosis; iron-dependent phospholipid peroxidation with impaired glutathione–GPX4 defense supports ferroptosis; and durable p16–RB or p53–p21 signaling supports senescence. IVDD studies have examined many of these nodes separately, but direct time-resolved comparisons under matched hypoxia or energy stress are rare. The available data therefore support shared upstream determinants and pathway crosstalk rather than a deterministic hierarchy of cell-fate switching [34,61,76,96,97,98,103,104,105,113,114,115,116,117,118,119,120,121,122,123,124,125,126,127,128,129,130,131,132].

Future experiments should combine time-resolved measurements of membrane potential, ATP, lysosomal function and mtROS with execution-specific assays and pathway-specific rescue. Cytochrome-c release and caspase activation, gasdermin cleavage and membrane permeabilization, lipid peroxidation and GPX4 status, phosphorylated MLKL, and durable senescence markers should be assessed in the same model. Single-cell or longitudinal approaches would be particularly useful for separating mixed populations from genuine transitions within individual cells. Until such evidence is available, the review treats the cell-fate programs as mechanistically connected but not interchangeable.

## 6. Consequences for Intervertebral Disc Homeostasis

Mitochondrial injury extends from the organelle to the whole disc through several connected processes. Loss of viable NP cells reduces aggrecan and collagen II synthesis. Senescent and inflammasome-active cells release cytokines and proteases that increase MMP and ADAMTS activity. mtROS and mitochondrial nucleic acids maintain NF-κB, NLRP3, TLR9 and cGAS–STING signaling, while macrophage–NP-cell crosstalk can amplify these responses [134]. Matrix turnover consequently shifts towards catabolism, reducing osmotic swelling and redistributing mechanical stress to the AF and CEP.

Surviving cells may also become dysfunctional before overt death. BNIP3 loss alters mitochondrial morphology, glycolytic capacity, ATP production and broader substrate use, and Bnip3-deficient mice show early NP degeneration despite compensatory mitophagy [17]. NLRX1 deficiency similarly uncouples selective fission from clearance and promotes metabolic collapse and senescence [19]. Mitophagy-dependent reprogramming of lactate metabolism and SPP1–integrin-mediated blockade of PINK1–Parkin further connect mitochondrial state to matrix phenotype and calcification [135,136]. Mitochondrial quality therefore determines whether living disc cells retain an appropriate metabolic and matrix-producing phenotype.

Endplate failure creates another route for tissue-level amplification. CEP-cell apoptosis, ferroptosis and calcification reduce permeability and restrict nutrient diffusion. NP and inner-AF cells are then exposed to greater glucose limitation, acidity and waste accumulation, which further compromise lysosomal and mitochondrial function. Nrf2/HO-1–Parkin signaling in CEP models may therefore preserve the central disc indirectly [8,9]. Direct experiments that trace these effects between disc regions remain rare.

Evidence that pathway manipulation changes disc structure comes from bovine organ culture and multiple needle-puncture, compression or instability models. Interventions that reduce mtROS, restore flux or enhance selected mitophagy pathways commonly preserve MRI signal, disc height, histological organization or matrix markers. Yet these models impose acute injury on young animals and do not reproduce decades of human ageing, endplate vascular change, immune exposure or pain biology. Human evidence is mainly cross-sectional, showing altered pathway regulators across degeneration grades. It supports disease relevance but cannot distinguish cause, compensation and consequence.

These cellular and tissue changes can form a reinforcing loop in experimental settings in which mitochondrial stress is prominent. Mechanical or nutritional stress may injure mitochondria; incomplete or maladaptive clearance can then sustain mtROS, cell loss, senescence and matrix catabolism, while structural deterioration further increases mechanical and metabolic stress. Other trajectories can begin with AF structural failure, CEP calcification, matrix defects or age-related proteoglycan loss without demonstrable mitophagy failure. Mitochondrial quality control may amplify or modify such trajectories after they begin. Mitochondria-directed treatment should therefore be matched to a documented mitochondrial defect rather than assumed to apply to all forms or stages of IVDD.

## 7. Current Potential Therapies

No intervention that jointly controls mtROS and mitophagic quality has been established as a disease-modifying treatment for human IVDD. All strategies discussed below remain preclinical.

**Figure 3:**
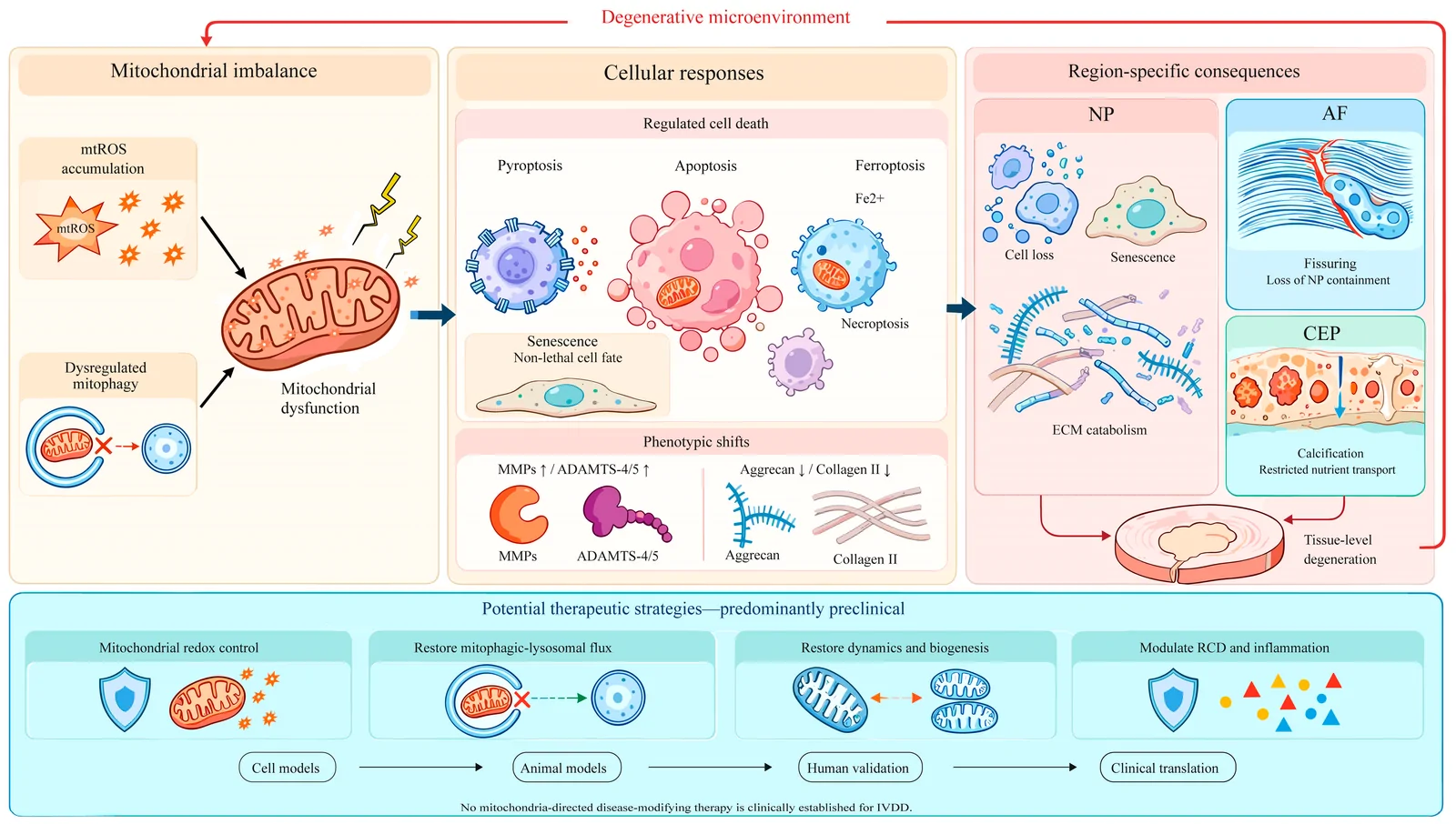
Context-dependent mitochondrial contributions to cell fate, regional disc pathology and potential intervention. mtROS accumulation and dysregulated mitophagy can contribute to mitochondrial dysfunction, cell-fate change and matrix catabolism. The relative sizes of the cell-fate illustrations represent the breadth of IVDD evidence reviewed, not biological prevalence or effect size: apoptosis has the broadest mechanistic literature; pyroptosis and ferroptosis have growing but model-dependent support; and direct mitophagy–necroptosis evidence is comparatively sparse. Senescence is shown separately because it is a non-lethal cell fate. The larger NP panel reflects the predominance of NP-derived studies; AF and CEP panels emphasize structural containment and nutrient-transport consequences without implying equivalent mitochondrial evidence. Candidate interventions are preclinical strategy classes, and the lower sequence indicates validation still required rather than completed clinical progression. ADAMTS, a disintegrin and metalloproteinase with thrombospondin motifs; AF, annulus fibrosus; CEP, cartilage endplate; ECM, extracellular matrix; IVDD, intervertebral disc degeneration; MMP, matrix metalloproteinase; mtROS, mitochondrial reactive oxygen species; NP, nucleus pulposus; RCD, regulated cell death. Solid arrows indicate proposed directional relationships, and the red return arrow denotes a feed-forward degenerative loop. Box colors are used for visual grouping and do not represent quantitative effect size.

Therapeutic evidence should be separated into two categories. Relatively direct evidence requires pathway-specific genetic perturbation or rescue together with mitochondrial cargo or flux measurements. By contrast, many antioxidants, natural products and biomaterials are pleiotropic, and altered mitophagy is only one of several associated effects. The latter studies support therapeutic hypotheses but do not establish mitophagy as the causal mediator. No clinical trial has yet validated a mitochondria-directed or mitophagy-directed disease-modifying treatment for IVDD. The context-dependent links among mitochondrial imbalance, cell fate, regional disc pathology and potential intervention strategies are summarized in Figure 3.

Mitochondrial redox control offers the most direct way to reduce the initial oxidative burden [7,133,137,138,139]. MitoQ lowers mtROS and preserves mitochondrial function in compressed NP cells and one-strike whole-disc culture [18,61]. Honokiol activates AMPK–PGC-1α–SIRT3 signaling, improves antioxidant capacity and mitochondrial dynamics, and attenuates puncture-induced degeneration in rats [23]. Melatonin has been linked to both Parkin-dependent clearance and PGC-1α-mediated coordination of mitochondrial biogenesis, dynamics and mitophagy [110,140]. These agents have several targets, so their benefits cannot be attributed solely to mitophagy.

Lowering mtROS alone does not remove damaged mitochondria. Restoring lysosomal competence and downstream flux may provide a complementary approach [81,82,83,123,124]. MitoQ improves mitophagosome–lysosome fusion during compression [18], while curcumin restores general autophagic flux through AMPK–mTOR–ULK1 signaling and improved lysosomal function [99]. Hypoxia- or oxidative-stress-preconditioned stromal-cell exosomes deliver cargo that enhances mitochondrial clearance [141,142]. Salidroside similarly restores SIRT1/FOXO3-dependent flux in NP-derived progenitors [39]. Delivery, cargo consistency and long-term safety remain unresolved for these strategies.

Greater selectivity may be achieved by targeting mitochondrial dynamics, cargo recognition or receptor-mediated clearance. NLRX1 restoration improves selective fission and prevents compensatory overclearance [19]. Inhibition of excessive ERK1/2–Drp1 or Piezo1–CaMKII–Drp1 activity may reduce widespread fragmentation [75,76]. FGF21/SIRT1/FOXO3 signaling and modulation of OPTN, FUNDC1, BNIP3 or PHB2 are also plausible targets [143]. Their effects are likely to vary with cell type and stress because mitophagy receptors overlap in function and can participate in pro-death responses.

Regional biology also constrains therapeutic interpretation. NP-directed studies chiefly address redox burden, lysosomal completion and mitochondrial mass under hypoxia and acidity. AF studies include direct evidence for regional metabolism [35,36], oxidative-stress-associated ferroptosis [37] and an engineered-exosome repair strategy linked to mitophagy recovery [38], but this literature is much smaller than the NP literature and does not justify transferring NP targets directly to AF cells. CEP-directed strategies must consider calcification, ferroptosis and nutrient transport as well as mitochondrial redox control [8,9,10,11,12,13]. Delivery studies should therefore identify the intended disc region and demonstrate local exposure and response rather than relying only on whole-disc histology.

When mitochondrial recovery remains incomplete, inflammatory and cell-death pathways become additional therapeutic targets [68,69,113,114,122,123,124]. Inhibition of NLRP3, ferroptosis or RIPK1–RIPK3–MLKL signaling may preserve disc cells. Blocking death execution without restoring mitochondrial fitness, however, could leave metabolically impaired or senescent cells in the tissue. Combination treatment may therefore need to reduce mtROS, restore competent flux and restrain the cell-death pathway that remains active.

Clinical translation is limited by both biology and delivery. The avascular, dense and acidic disc restricts penetration, while drug retention varies with molecular properties and delivery vehicle [144]. Intradiscal puncture can also create a leakage path; through-puncture experiments show that leakage depends on puncture and injectate conditions, so retention cannot be inferred from injection alone [145]. Systemic mitochondrial targeting raises off-target concerns, whereas repeated local injection may damage AF containment. Biological dose is equally important; sustained ROS suppression may blunt redox signaling, and indiscriminate mitophagy enhancement may remove functional mitochondria from cells with limited renewal capacity.

Hydrogels and nanoparticles offer delivery solutions, not proof of clinical efficacy. Injectable hydrogels can prolong local residence and provide controlled release; nanoparticles can protect cargo or support mitochondrial and stimulus-responsive targeting [90,93,114,133,144,146,147]. An AF-repair microneedle/exosome system provides region-specific proof of concept for combining structural repair with modulation of mitophagy [38]. These platforms still require assessment of diffusion through the disc matrix, leakage, degradation products, sterilization, manufacturing consistency, long-term biocompatibility and performance under physiological loading.

Patient selection is equally important. A strategy designed for excessive mtROS may not correct lysosomal failure, and a mitophagy enhancer may be unsuitable when mitochondrial replacement is already limited. Future studies should define the dominant defect, the intended disc region and the degeneration stage before assigning therapeutic readiness. Table 3 therefore separates target-level mechanistic support from pleiotropic association and delivery proof-of-concept.

**Table 3:** Preclinical therapeutic strategies targeting mitochondrial dysfunction and mitophagy in IVDD.

Strategy	Representative Interventions	Target and Main Regional Focus	Mechanistic Evidence	Translational Limitation	Representative Studies
Mitochondrial redox control	MitoQ, honokiol, melatonin and selenium nanoparticles	mtROS and antioxidant defence; mainly NP, with selected whole-disc and CEP evidence	Mostly pleiotropic association; redox rescue is clearer than mitophagy causality	Pleiotropic targets, dosing and long-term safety	Kang et al. [18]; Wang et al. [23]; Song et al. [46]; Chen et al. [110]; He et al. [137]; Lan et al. [140]
Restore mitophagic and lysosomal flux	Curcumin, salidroside, taurine, urolithin A and preconditioned BMSC exosomes	Mitophagosome–lysosome completion; mainly NP cells and NP progenitors	Mixed evidence; some flux rescue, but many interventions have multiple targets	Delivery, vesicle-cargo consistency and durability	Li et al. [39]; Lin et al. [91]; Lin et al. [92]; Kang et al. [99]; Jin et al. [141]; Teng et al. [142]
Improve cargo selection and receptor signalling	NX-13/NLRX1, FBXO2, FGF21 and PHB2	Selective fission, cargo recognition and lysosomal delivery; predominantly NP evidence	Relatively direct target-level genetic or pathway-manipulation evidence	Context-dependent pathway effects and no clinical dosing data	Song et al. [19]; Hu et al. [32]; Gu et al. [33]; Zhu et al. [34]; Wu et al. [126]; Wu et al. [143]
Modify dynamics and proteostasis	Drp1 inhibition, MANF and ATF5	Mitochondrial dynamics and proteostasis; NP evidence with selected AF and CEP studies	Mixed target-level evidence linking dynamics or proteostasis to mitochondrial outcomes	Off-target effects and uncertain repair–disposal balance	Chen et al. [30]; Xu et al. [51]; Xu et al. [52]; Ke et al. [62]; Hu et al. [73]; Ma et al. [112]
Limit downstream injury signalling	Humanin, taurine, scutellarin and HSP90 inhibition	mtDNA, NLRP3 and death execution; mainly NP, with selected CEP ferroptosis evidence	Mostly downstream or pleiotropic evidence; mitochondrial fitness may remain impaired	Damaged or senescent cells may persist if mitochondrial fitness is not restored	Zhu et al. [96]; Zai et al. [97]; Hu et al. [109]; Wang et al. [103]; Zhao et al. [104]; Zhang et al. [113]
Local delivery and combination strategies	Hydrogels, nanoparticles, responsive scaffolds and AF-repair microneedle/exosome systems	Local retention or structural repair; NP-centred delivery with emerging AF-specific evidence	Delivery proof-of-concept in preclinical models, not evidence of clinical readiness	Injection leakage, diffusion, manufacturing, durability and regional exposure	Wu et al. [90]; Zhang et al. [93]; Chen et al. [146]; Gao et al. [147]; Hu et al. [38]; Rudnik-Jansen et al. [144]; Varden et al. [145]

All interventions remain preclinical, and none has been established as a disease-modifying treatment for human IVDD. “Relatively direct” evidence denotes target-specific perturbation or rescue supported by flux or mitochondrial-cargo measurements; “pleiotropic” evidence denotes concurrent mitophagy-marker changes among several biological effects. Regional applicability is inferred mainly from NP studies unless AF or CEP evidence is explicitly stated. AMPK, AMP-activated protein kinase; ATF5, activating transcription factor 5; BMSC, bone-marrow-derived mesenchymal stromal cell; CEP, cartilage endplate; Drp1, dynamin-related protein 1; FGF21, fibroblast growth factor 21; HSP90, heat shock protein 90; IVDD, intervertebral disc degeneration; NP, nucleus pulposus; NLRP3, NLR family pyrin domain-containing 3; NLRX1, NLR family member X1; PHB2, prohibitin 2; SIRT1, sirtuin 1.

## 8. Conclusions

Current evidence does not support a simple sequence in which ROS cause injury and mitophagy provides protection, nor does it support mitophagy failure as a universal initiating cause of IVDD. Disc degeneration may progress through AF structural compromise, CEP calcification, matrix abnormalities and age-related proteoglycan loss without demonstrated failure of mitochondrial clearance. Within mitochondrial-stress-dominant contexts, however, a capacity-matching model helps explain why the same pathway can be protective, ineffective or maladaptive. Mismatch can arise from blocked lysosomal flux, non-selective fragmentation, clearance that exceeds biogenesis or failure of the remaining network to meet regional metabolic demand. Selective MDV transport may remove mitochondrial material before whole-organelle clearance becomes necessary, whereas TDP43-containing mitochondria-derived vesicles in NP cells may propagate stress between cells; their relationship to lysosome-directed MDVs remains unresolved [60].

The strength of the evidence differs among cell fates. Mitochondrial apoptosis and senescence are supported by human-cell studies, organ culture and in vivo models. Mechanistic evidence for pyroptosis and ferroptosis is growing, whereas necroptosis remains less developed and mixed execution states are often poorly resolved. Increased PINK1, Parkin, LC3 or mitochondrial turnover should therefore be interpreted only after cargo selectivity, lysosomal completion, mitochondrial replacement and the relevant execution pathway have been examined.

Progress depends on measuring mitochondrial clearance rather than inferring it from PINK1, Parkin, LC3 or autophagosome abundance. Tandem mCherry–GFP–LC3 reports general autophagosome maturation and is useful for detecting lysosomal blockade, but it does not identify mitochondrial cargo. Mitochondria-specific reporters, such as mito-QC or mt-Keima, together with lysosomal-inhibitor controls and colocalization of mitochondrial cargo with lysosomes, are more appropriate for mitophagic flux [22,148,149]. Flux measurements should be paired with mitochondrial mass, respiration or ATP production and indices of biogenesis so that clearance can be interpreted against replacement. Regional comparisons should use NP, AF and CEP cells under relevant oxygen, pH and loading conditions.

Longitudinal designs should connect these mitochondrial states to execution-specific cell-death assays and matrix outcomes. MDV studies should determine how intracellular and extracellular destinations are selected and whether vesicle release increases when mitophagic or lysosomal capacity is exceeded. The capacity-matching framework is therefore an IVDD-specific conceptual synthesis, not a newly discovered molecular pathway. Its value lies in defining measurable conditions that may explain why the same mitophagy-associated intervention is protective in one disc context but ineffective or harmful in another.

## 9. Literature Search Strategy

This work was designed as a structured narrative review rather than a systematic review or meta-analysis. PubMed/MEDLINE was searched for publications from 1 January 2012 to 11 September 2026 (inclusive). For articles published online ahead of issue assignment, the first online publication date was used to determine eligibility. The final date-limited query was rerun through the PubMed interface on 16 October 2026 and returned 1137 records; this rerun verified the search expression and record count and did not extend the prespecified evidence cutoff:

((“Intervertebral Disc Degeneration”[Mesh] OR “intervertebral disc degeneration”[tiab] OR IVDD[tiab] OR “degenerative disc disease”[tiab] OR “nucleus pulposus”[tiab] OR “annulus fibrosus”[tiab] OR “cartilage endplate”[tiab]) AND (“Mitophagy”[Mesh] OR mitophagy[tiab] OR “Mitochondria”[Mesh] OR mitochondria*[tiab] OR “mitochondrial dysfunction”[tiab] OR “Reactive Oxygen Species”[Mesh] OR “mitochondrial reactive oxygen species”[tiab] OR mtROS[tiab] OR “Oxidative Stress”[Mesh] OR “oxidative stress”[tiab] OR “mitochondrial-derived vesicle”[tiab] OR “mitochondrial-derived vesicles”[tiab] OR MDV[tiab] OR MDVs[tiab])) AND (“2012/01/01”[Date—Publication]: “2026/09/11”[Date—Publication]).

Section-specific searches added the terms “regulated cell death”, apoptosis, pyroptosis, ferroptosis, necroptosis, senescence, PINK1, Parkin, BNIP3, FUNDC1, OPTN, PHB2 and NLRX1. Google Scholar and reference lists were also screened. Earlier publications were retained only when needed to establish foundational AF mechanobiology or flux-reporter methods that could not be replaced by a later primary source; these citation-tracked exceptions are explicitly distinguished from the 15-year primary search window.

Eligible sources included English-language primary studies using human disc tissue, disc-derived cells, organ culture or animal IVDD models that examined mitochondrial redox stress, mitophagy, MDVs or regulated cell fate. Selected mechanistic studies outside the disc field were retained only to define MDV biology or a measurement method. Studies unrelated to disc biology, reports without relevant mitochondrial outcomes, conference abstracts, duplicate publications and non-English reports were excluded. Reviews were used for context but not as substitutes for primary evidence when original studies were available.

Source selection was iterative and was based on relevance to the review questions, model provenance and mechanistic informativeness. This was not a systematic review: duplicate independent screening, a PRISMA flow diagram, prospective protocol registration, formal risk-of-bias scoring and a complete exclusion ledger were not used. Accordingly, Table 2 provides descriptive information on the kinds of evidence and their principal limitations rather than a formal GRADE-style hierarchy. Earlier publications were retained only for foundational AF mechanobiology or flux-reporter methods that could not be replaced by later primary studies.

## Data Availability

No new data were created or analyzed in this study. Data sharing is not applicable to this article.
